# Early Postnatal Hypocapnia and Hypercapnia in Ventilated Preterm Infants: Incidence and Associations with Adverse Outcomes

**DOI:** 10.3390/jpm16040212

**Published:** 2026-04-12

**Authors:** Ilias Chatziioannidis, Angeliki Kontou, Eleni Agakidou, Theodora Stathopoulou, Kostantia Tsoni, Christos Paschaloudis, William Chotas, Kosmas Sarafidis

**Affiliations:** 1 1st Department of Neonatology and Neonatal Intensive Care, Faculty of Medicine, School of Health Sciences, Ippokrateion General Hospital, Aristotle University of Thessaloniki, Kostantinoupoleos 49 Str., 54642 Thessaloniki, Greece; ihatzi@auth.gr (I.C.); angiekon2001@yahoo.gr (A.K.); eagakidou@auth.gr (E.A.); dorastatho@gmail.com (T.S.); ntina.tsoni@gmail.com (K.T.); chrispashaloudis@hotmail.com (C.P.); 2 Department of Neonatology, Memorial Hospital of Carbondale, Southern Illinois Healthcare, Carbondale, IL 62901, USA

**Keywords:** neonate, carbon dioxide, mechanical ventilation, personalized medicine

## Abstract

**Background/Objectives:** Abnormalities in the partial pressure of carbon dioxide (PCO_2_) can occur during respiratory support and may contribute to adverse neonatal outcomes. This study aimed to assess the incidence of early hypocapnia and hypercapnia in mechanically ventilated preterm infants and their major associated outcomes. **Methods:** A single-center retrospective cohort study (2017–2024) was conducted in preterm infants < 32 weeks’ gestation who required > 24 h of invasive ventilation within the first 3 days of life. Perinatal–neonatal data were retrieved from the medical database. Admission blood gas values (arterial and capillary–venous) and the maximum and minimum PCO_2_ in the first 72 h were evaluated. Normocapnia was defined as PCO_2_ 35–45 mmHg, hypocapnia as < 35 mmHg, and hypercapnia as > 45 mmHg. Primary outcomes were the incidence of PCO_2_ abnormalities; secondary outcomes included death or severe brain injury (SBI), SBI alone, and bronchopulmonary dysplasia (BPD) among survivors. Logistic regression identified independent predictors of the secondary outcomes. **Results:** Among the 134 infants evaluated, most experienced both hypercapnia and hypocapnia. Hypercapnia occurred in 81.3% of infants, and hypocapnia in 93.2%. Death or SBI was observed in 51.5%, and SBI alone in 42.5%. Gestational age < 28 weeks, air-leak syndromes, and pulmonary hemorrhage were independent predictors of death or SBI. Among survivors, hypercapnia and gestational age < 28 weeks independently predicted BPD. Infants with adverse outcomes had higher maximum PCO_2_ values and greater PCO_2_ variability, although these were not independent predictors of SBI or death. **Conclusions:** PCO_2_ instability is highly prevalent in ventilated preterm infants, underscoring the need for individualized ventilation strategies. Extreme prematurity emerged as the primary risk factor for adverse outcomes, while hypercapnia was independently associated with BPD.

## 1. Introduction

Over the past few decades, survival rates for preterm infants have improved markedly [1]. Additionally, modern practice emphasizes non-invasive respiratory support to reduce complications associated with mechanical ventilation. Nevertheless, many preterm infants with respiratory distress syndrome still require invasive ventilation [1,2,3]. In this context, maintaining a normal partial pressure of carbon dioxide (PCO_2_) in the blood is essential for preserving acid–base balance, yet optimal PCO_2_ targets remain unclear [4].

Hypocapnia and hypercapnia are common in neonatal intensive care units (NICUs) [5], and often reflect underlying illness or aspects of respiratory management, such as inappropriate iatrogenic hyperventilation [6] or intentional permissive hypercapnia [7].

Accumulating evidence, however, suggests that avoiding both hypocapnia and hypercapnia is crucial, as extreme deviations and wide fluctuations in PCO_2_ levels are well-recognized risk factors for serious complications. These complications include neurological injuries such as intraventricular hemorrhage (IVH) and periventricular leukomalacia (PVL) [8,9,10]. In addition, an association between hypocarbia and the development of bronchopulmonary dysplasia (BPD) has been found [11]. Notably, permissive hypercapnia, aimed at minimizing ventilator-induced lung injury, has been suggested as a promising neonatal ventilation strategy [7]. However, the recent literature shows no significant differences in the prevalence of BPD or death with permissive hypercapnia, while a potential association with an increased risk of necrotizing enterocolitis (NEC) remains a concern [12,13].

Τhe objective of the present cohort study in ventilated preterm infants was twofold: (a) to determine the incidence and patterns of PCO_2_ abnormalities (hypocapnia, hypercapnia, and variability) during the first 72 h of life, and (b) to evaluate the associations between these PCO_2_ deviations and major neonatal outcomes, including survival, severe brain injury (SBI) and BPD. By investigating early PCO_2_ disturbances and their associations with key neonatal outcomes, clinically relevant insights may be gained that will support optimal neonatal medical care and promote a personalized medical approach, enabling ventilation strategies to be individually tailored to the specific condition and medical needs of each preterm infant.

## 2. Materials and Methods

### 2.1. Patients and Data Collection

We conducted a single-center retrospective cohort study (2017–2024) of preterm infants (<32 weeks’ gestational age [GA]) who required invasive ventilation for >24 h within the first 3 days of life. Perinatal and neonatal data were obtained from our center’s medical database. This allowed for the evaluation of blood gas values (pH, PCO_2_, base deficit) at NICU admission, as well as the maximum and minimum PCO_2_ values during the first three days. The difference between these two values was calculated as an indicator of fluctuation. As in other studies [13,14], arterial and capillary–venous samples were not distinguished because many infants lacked arterial access. Exclusion criteria included major congenital anomalies, advanced resuscitation at birth, death within 24 h, and insufficient clinical data.

### 2.2. Outcomes and Definitions

Primarily, we aimed to evaluate the incidence of normocapnia, hypocapnia, and hypercapnia. Secondarily, we aimed to identify any associations between these PCO_2_ abnormalities and important neonatal outcomes, including the combined outcome of death prior to NICU discharge or SBI (defined as IVH grades III–IV or cystic PVL), SBI alone, and BPD among survivors with or without SBI.

Normocapnia was defined as a PCO_2_ of 35–45 mmHg, hypocapnia as a PCO_2_ < 35 mmHg, and hypercapnia as a PCO_2_ > 45 mmHg, in accordance with thresholds commonly used in neonatal research [4]. SBI was defined as the presence of IVH grades III–IV or cystic PVL according to specific ultrasound diagnostic criteria [15,16] and BPD was defined as the need for oxygen therapy or mechanical ventilation at 36 weeks postmenstrual age [17].

### 2.3. Respiratory Management

Although significant changes in respiratory management occurred in our NICU during the study period, we typically prioritized non-invasive ventilation and less invasive surfactant administration for respiratory distress syndrome (RDS) in infants ≥ 24 weeks’ gestation. When invasive mechanical ventilation was required, exogenous surfactant was administered via the endotracheal tube. Starting in 2020, we implemented synchronized positive pressure ventilation with volume guarantee (VG) as the primary mode of invasive ventilation for preterm neonates. Respiratory care was escalated to rescue high-frequency oscillatory ventilation (HFOV) after failure of conventional ventilation. During the acute phase of RDS, we targeted blood gas PCO_2_ values of 35–50 mmHg and pH ≥ 7.2 and attempted to maintain oxygen saturation between 90 and 95%.

### 2.4. Statistical Analysis

Continuous variables were summarized as the mean ± standard deviation when approximately normally distributed, or as median with first and third quartiles (Q1, Q3) otherwise. Categorical variables were summarized as counts and percentages. Normality was assessed using the Shapiro–Wilk test and Q–Q plots. For group comparisons we used either Student’s *t*-test or the Mann–Whitney test, as appropriate in each case for continuous variables, and the Chi-square test or Fisher’s exact test when cell counts were <5 for categorical variables.

To describe the associations for each of the outcomes (death or SBI, SBI and BPD for survivors) with the possible predictors, we fitted 3 logistic regression models. Final models are presented as odds ratios (ORs) with 95% confidence intervals and *p*-values. The log-likelihood test and Akaike Information Criterion (AIC) values were used for comparison and selection between models. Model performance was evaluated using the area under the ROC curve with 95% confidence intervals. The significance level was set at 0.05 for all tests. Statistical analysis was performed using the statistical program R 4.4.1.

## 3. Results

During the study period, 351 neonates < 32 weeks’ gestation were admitted to our NICU, of whom 169 required intubation and mechanical ventilation for more than 24 h during the first 3 days of life. After applying the exclusion criteria, 134 infants were included in the final analysis.

### 3.1. Study Population Characteristics

Data on perinatal and neonatal characteristics of the study population are provided in Table 1 along with important clinical outcomes involving survivors only, such as BPD and retinopathy of prematurity (ROP).

### 3.2. Variability in PCO_2_ During the First 72 h

In our cohort, most infants experienced both hypercapnia and hypocapnia (Table 2), reflecting wide intra-individual variability in PCO_2_ values. Hypercapnia occurred in 109 infants (81.3%), while the PCO_2_ remained at 45 mmHg or less in 25 infants (18.7%). Hypocapnia was even more common, and was observed in 125 infants (93.2%), with 87 (64.9%) developing severe hypocapnia (PCO_2_ < 30 mmHg). Notably, severe hypocapnia was often associated with concurrent episodes of moderate-to-severe hypercapnia (PCO_2_ > 55 mmHg).

### 3.3. Outcomes

#### 3.3.1. Death or Severe Brain Injury

As shown in Table 3, infants who died or developed SBI had significantly lower GA and birth weight and were significantly more likely to be born before 28 weeks compared to survivors without SBI. They also had significantly lower 5 min Apgar scores and experienced significantly more complications, including pulmonary hemorrhage, air-leak syndromes, treated PDA, and NEC grade II–III. Moreover, these infants more often required rescue HFOV and exhibited greater metabolic acidosis, higher and more variable PCO_2_ levels, and a higher incidence of hypercapnia.

#### 3.3.2. Severe Brain Injury

Infants who developed SBI had significantly lower GA and birth weights and were more often male compared to those without SBI. They also experienced significantly higher rates of complications, including pulmonary hemorrhage, air-leak syndromes, treated PDA, and exposure to sedatives. HFOV was used significantly more often in this group, and survival was markedly lower. In addition, infants with SBI exhibited significantly higher maximum PCO_2_ levels and greater PCO_2_ fluctuations (Online Supplementary Material Table S1).

#### 3.3.3. Survivors with and Without BPD

Among survivors, infants who developed BPD had significantly lower GA and birth weight, and extremely preterm birth (<28 weeks) was much more common in this group. They also experienced significantly higher rates of treated PDA and ROP, were more frequently exposed to sedatives, and required rescue HFOV more often. In addition, these infants had longer durations of invasive ventilation and longer hospital stays. Early PCO_2_ instability was more pronounced in infants with BPD, as reflected by higher maximum PCO_2_ levels and more frequent hypercapnia, while other blood gas parameters were similar between groups (online Supplementary Material Table S2).

### 3.4. Multivariable Analyses

We constructed three multiple logistic regression models to evaluate risk factors for (1) death or SBI, (2) SBI independent of death, and (3) BPD among survivors in very preterm neonates (Table 4). GA was included as a binary variable (<28 weeks vs. ≥28 weeks).

**Model 1:** GA < 28 weeks was the strongest predictor of death or SBI, significantly increasing the odds of these outcomes. Respiratory complications, including air-leak syndromes and pulmonary hemorrhage, were also independently associated with higher risk. Absolute base deficit was an additional significant predictor in the model. The area under the ROC curve (AUC) was 0.895 (95% CI 0.840–0.949).

**Model 2:** When SBI independent of death was analyzed, the results were largely similar to those for death or SBI. Extremely preterm neonates remained at markedly higher risk, and respiratory complications, including air-leak syndromes and pulmonary hemorrhage, continued to be significant predictors. Male sex also emerged as an independent risk factor for SBI. The AUC was 0.895 (95% CI 0.840–0.948).

**Model 3:** Among survivors, GA < 28 weeks was significantly associated with increased odds of BPD. PDA requiring treatment showed a trend toward higher risk, and hypercapnia was independently associated with BPD. The AUC was 0.808 (95% CI 0.718–0.897).

## 4. Discussion

In this cohort study, PCO_2_ instability was common among preterm infants < 32 weeks’ gestation requiring mechanical ventilation, with many experiencing both hypocapnia and hypercapnia during the first three days of life. These abnormalities were more frequent in infants who developed adverse outcomes, including death, SBI, and BPD. Although extreme prematurity emerged as the primary contributing factor to these outcomes, hypercapnia was independently associated only with BPD.

Few studies have examined the incidence of hypocapnia and hypercapnia, particularly their co-occurrence, in mechanically ventilated infants. In the large European Neovent cohort (2013), 4% of infants receiving invasive respiratory support had hypocapnia (PCO_2_ < 30 mmHg) and 31% had hypercapnia (PCO_2_ > 52 mmHg). Moreover, hypocapnia occurred most often during the first three days of life, whereas hypercapnia became more prevalent after one week [5]. Subsequent research in ventilated very-low-birth-weight infants also revealed frequent episodes of both PCO_2_ abnormalities with inadequate staffing linked to higher rates of BPD [18]. In addition, even after the implementation of a neuroprotection care bundle, which included interventions aimed at optimal respiratory management, the incidence of hypocapnia (<35 mmHg), although reduced from 43.9% to 34.6%, remained common during the first 72 h, while hypercapnia (>60 mmHg) changed a little (38.5% vs. 34.1%) [19]. Another cohort of infants < 32 weeks reported <2% hypocapnia and mild, moderate, and severe hypercapnia had prevalences of 26.5%, 13%, and 6.5%, respectively [8]. Finally, a single-center study of 100 ventilated neonates documented hypocapnia in 82% and hypercapnia in 77%, although overlap within individual infants was not assessed [6]. These studies demonstrate the difficulty in maintaining normocapnia and are consistent with the PCO_2_ variability also observed in our study.

Both hypocapnia and hypercapnia have been associated with increased mortality and significant morbidities, with invasive respiratory support being a significant contributing factor. In an exploratory analysis of a randomized trial, hypercapnic extremely low-birth-weight infants were found to have significantly increased mortality and required higher respiratory support, each reflecting disease severity. Thus, although elevated CO_2_ levels appear to be associated with poorer outcomes, they likely reflect illness severity rather than being a direct cause of complications [20]. A single-center study reported that 82% of mechanically ventilated neonates experienced hypocapnia, with mortality reaching 67.1% [6]. Hypocapnia has long been recognized to induce cerebral vasoconstriction and decrease cerebral blood flow, increasing the risk of cerebral palsy and long-term neurodevelopmental impairment [21]. Conversely, hypercapnia causes cerebral vasodilation and hyperperfusion, increasing the risk of cerebral edema, IVH, and, at very high levels (>60 mmHg), seizures and decreased consciousness [22]. Current data indicate that hypercapnia may cause injury to the cerebral cortex and lead to cognitive deficits in newborn piglets [23].

The recent (2022) systematic review by Wong et al. summarized the accumulated evidence on PCO_2_ levels in preterm infants [4]. Hypocapnia was consistently associated with adverse neurological outcomes, with greater or more prolonged exposure to low PCO_2_ conferring a higher risk of poor outcomes, predominantly neurological, and in some settings, increased mortality or severe disability. Hypercapnia, on the other hand, apart from the adverse neurological outcomes (IVH), was associated with other important morbidities of prematurity such as BPD, NEC, and ROP. Notably, the 2025 European consensus guidelines on the management of RDS suggest that modest hypercarbia is reasonable and that PCO_2_ levels around 5–7 kPa (≈38–53 mmHg) are likely optimal, if the pH remains above 7.22 [24]. The recommended PCO_2_ ranges are based on the systematic reviews by Wong et al. [4] and Ozawa et al. [12], whereas the pH threshold likely reflects expert panel consensus. This appears to lie between the pH thresholds of ≥7.20 and ≥7.25 in the higher and lower hypercapnia arms, respectively, as reported by Travers et al. [13]. No pre-specified minimum pH has been described in other relevant studies of permissive hypercapnia [25].

Large database studies offer valuable context for neonatal outcomes, but they rarely focus solely on preterm infants receiving invasive respiratory support [26,27,28]. In contrast, we analyzed only mechanically ventilated infants, predominantly with RDS, and assessed PCO_2_ in relation to death or SBI. As expected, extreme prematurity (23.1% born at 22–24 weeks) was the strongest independent predictor of death or SBI alongside pulmonary complications (air-leak, pulmonary hemorrhage) and metabolic acidosis (as indicated by base deficit). These findings support the hypothesis that fluctuations in cerebral perfusion and PCO_2_ impair autoregulation and heighten vulnerability to hemorrhagic and ischemic brain injury, even though maximum PCO_2_ and variability were not independent predictors in our multivariable analysis. Overall, our study showed that individual infant vulnerability early after birth, driven by extreme prematurity and its complications, was a more important contributor to death or SBI than single PCO_2_ derangements.

BPD remains a major complication of prematurity. In our cohort, survival with BPD was associated with extreme prematurity, prolonged ventilation, and complications such as hemodynamically significant PDA requiring treatment. Moreover, infants with BPD exhibited higher maximum PCO_2_, greater variability, and more frequent hypercapnia. Multivariable analysis confirmed that both gestational age < 28 weeks and hypercapnia independently predicted BPD in survivors. Previous studies similarly implicated hypercapnia and wide CO_2_ fluctuations as potential contributors to BPD [11,29], although the association may reflect disease severity, permissive hypercapnia, or intrinsic immature lung characteristics [30]. Thus, irrespective of whether increased PCO_2_ is simply associated with or causally contributes to BPD, hypercapnia could be considered an early predictor of lung injury and disease progression.

Ventilation mode is well recognized to influence PCO_2_ control. In the Neovent cohort study, infants receiving pressure-limited ventilation had lower PCO_2_ compared with those receiving volume-targeted or high-frequency ventilation [5]. VG—a mode of volume-targeted ventilation that automatically adjusts peak inspiratory pressure to deliver a consistent tidal volume—is considered helpful in preventing both hyperventilation and hypoventilation, thereby reducing the risk of lung and brain injury. Several meta-analyses have shown that compared with conventional ventilation, volume-targeted modes significantly reduce BPD, duration of mechanical ventilation, pneumothorax, the combined outcome of death/BPD, and SBI [3]. As VG was widely implemented in our NICU only from 2020 onward, earlier adoption might have reduced the number of PCO_2_ abnormalities observed in our cohort. Additionally, half of the neonates in our study received rescue HFOV. However, HFOV was in most cases not combined with VG, the use of which might have resulted not only in improved oxygenation but also in fewer PCO_2_ abnormalities and survival with less severe BPD [31].

This study contributes to the limited evidence on the early incidence of hypocapnia and hypercapnia in ventilated preterm infants and their relationship with severe complications. Quantifying PCO_2_ abnormalities underscores the challenges of maintaining ventilation and oxygenation targets in sick neonates receiving invasive respiratory support [32,33].

Although our retrospective design limited serial blood gas monitoring (including pH recording) and differentiation between arterial and capillary–venous samples, the first three days of life remain a critical period before physiological compensation, making PCO_2_ a meaningful marker of early ventilatory status. Moreover, the availability of only a single minimum and maximum PCO_2_ value may have led to an overestimation of the incidence of hypocapnia and hypercapnia and may have captured transient changes rather than sustained exposure. As this was a single-center study, NICU-specific practices may also have influenced the results, which limits generalizability to other settings. Data were collected over seven years, during which ongoing efforts to implement evidence-based practices aimed at reducing brain [34] and lung injury [35,36] were underway. These efforts included personalized care strategies for optimal PCO_2_ control (e.g., use of VG in conventional ventilation), enhanced monitoring such as continuous end-tidal CO_2_, and near-infrared spectroscopy to assess brain oxygenation and perfusion. In general, neonatal care bundles have been associated with reduced SBI [19,37], though their impact may be diminished as more extremely preterm infants, with an inherently high risk for IVH, receive intensive care [38]. Further research is needed to establish safe PCO_2_ ranges for different gestational ages, investigate long-term neurodevelopmental outcomes, and develop strategies to minimize PCO_2_ variability.

## 5. Conclusions

This cohort study found a high prevalence of early PCO_2_ instability in mechanically ventilated very preterm infants, with frequent exposure to both hypocapnia and hypercapnia. PCO_2_ abnormalities were more common in infants with adverse outcomes (death, SBI, and BPD), which were largely attributable to extreme prematurity. Only an independent association between hypercapnia and BPD could be demonstrated. These findings highlight the importance of close PCO_2_ monitoring to protect the vulnerable preterm brain and lungs, while supporting a personalized approach to neonatal ventilatory care, in which PCO_2_ management and targets are tailored to each infant’s gestational age, physiologic stability, and risk of adverse outcomes.

## Figures and Tables

**Table 1:** Perinatal and neonatal characteristics of the study population.

Variable	Descriptive Statistics
	(*n* = 134)
Gestational age (weeks)	28 (25–29)
Gestational age < 28 (weeks)	62 (46.3%)
Birth weight (g)	950 (700–1230)
5 min Apgar score	8 (7–8)
Male sex	63 (47.0%)
SGA	20 (14.9%)
Maternal hypertension	22 (16.4%)
Chorioamnionitis	28 (20.9%)
PPROM	35 (26.1%)
Prenatal steroids	116 (86.6%)
Mg administration	76 (56.7%)
Cesarean section	114 (85.1%)
Inborn	120 (89.6%)
Surfactant for RDS	123 (91.8%)
Pulmonary hemorrhage	31 (23.1%)
Air-leak syndromes	25 (18.7%)
PDA	66 (49.3%)
Treated PDA	54 (40.3%)
Severe IVH	39 (29.1%)
SBI	57 (42.5%)
Sepsis (culture-positive; early/late)	69 (51.5%)
Fentanyl or sedative	69 (51.5%)
NEC (grade II, III)	17 (12.7%)
Rescue HFOV	69 (51.5%)
Duration of invasive ventilation (days)	5.0 (2.0–11.0)
Survival	83 (61.9%)
Death or SBI	69 (51.5%)
Length of stay (days)	51 (11–85)
At NICU admission	
pH	7.3 (7.2–7.4)
PCO_2_	36.8 (30.1–44.0)
Base Deficit (absolute value)	6.8 (5.2–9.2)
During the first 3 days of life	
Min PCO_2_	28.0 (± 5.2)
Max PCO_2_	55.6 (±13.4)
Max–min PCO_2_	24.5 (16.4–33.4)
Clinical outcomes in survivors (*n* = 83)	
Any BPD	54 (65.9%)
ROP	33 (40.2%)
Treated ROP	13 (15.9%)
Length of stay (days)	75 (54–115)

Data are presented as mean (SD), median (Q1, Q3) or counts (%). BPD: bronchopulmonary dysplasia; CS: cesarean section; HFOV: high-frequency oscillatory ventilation; IVH: intraventricular hemorrhage; NEC: necrotizing enterocolitis; NICU: neonatal intensive care unit; Mg: magnesium; PDA: patent ductus arteriosus; PPROM: preterm premature rupture of membranes; RDS: respiratory distress syndrome; ROP: retinopathy of prematurity; SBI: severe brain injury; SGA: small for gestational age.

**Table 2:** Cross-tabulation of min and max PCO_2_ (mmHg) of the study population.

	Max PCO_2_ Group			
Min PCO_2_ Group	35–45	46–55	56–65	>65
**<30**	21 (15.7%)	27 (20.1%)	17 (12.7%)	22 (16.4%)
**30–34**	4 (3%)	19 (14.2%)	11 (8.2%)	4 (3%)
**35–45**	0 (0%)	5 (3.7%)	4 (3%)	0 (0%)

Values represent the number and percentage of participants within each PCO_2_ group.

**Table 3:** Perinatal and neonatal characteristics of infants who survived without SBI and of those with the combined outcome of death or SBI.

Variable	Survival Without SBI (*n* = 65)	Death or SBI (*n* = 69)	*p*-Value
Gestational age (weeks)	29 (28–30)	25 (24–27)	<0.001
Gestational age < 28 weeks	9 (13.8%)	53 (76.8%)	<0.001
Birth weight (g)	1170 (1020–1380)	710 (595–960)	<0.001
5 min Apgar score	8 (7–9)	7 (7–8)	0.006
Male sex	25 (38.5%)	38 (55.1%)	0.054
SGA	9 (13.8%)	11 (15.9%)	0.734
Maternal hypertension	14 (21.5%)	8 (11.6%)	0.120
Chorioamnionitis	10 (15.4%)	18 (26.1%)	0.128
PPROM	12 (18.5%)	23 (33.3%)	0.050
Prenatal steroids	55 (84.6%)	61 (88.4%)	0.520
Mg administration	35 (53.8%)	41 (59.4%)	0.515
Cesarean section	60 (92.3%)	54 (78.3%)	0.029
Inborn	60 (92.3%)	60 (87.0%)	0.401
Surfactant for RDS	58 (89.2%)	65 (94.2%)	0.356
Pulmonary hemorrhage	5 (7.7%)	26 (37.7%)	<0.001
Air-leak syndromes	7 (10.8%)	18 (26.1%)	0.023
Treated PDA	15 (23.1%)	39 (56.5%)	<0.001
Sepsis (culture-positive; early/late)	31 (47.7%)	38 (55.1%)	0.393
Fentanyl or sedative	29 (44.6%)	40 (58.0%)	0.122
NEC (grade II, III)	4 (6.2%)	13 (18.8%)	0.037
Rescue HFOV	19 (29.2%)	50 (72.5%)	<0.001
Duration of invasive ventilation	4 (1; 9)	7 (4; 15)	0.005
At NICU admission			
pH	7.3 (7.3; 7.4)	7.3 (7.2; 7.4)	0.540
PCO_2_	37.4 (31.3–47.0)	36.5 (29.4–42.6)	0.223
Base Deficit (absolute value)	5.9 (4.4–8.5)	7.4 (5.9; 9.8)	0.005
During the first 3 days of life			
Min PCO_2_	28.8 (± 5.3)	27.3 (± 5.0)	0.116
Max PCO_2_	51.2 (± 10.8)	59.8 (± 14.3)	<0.001
Max-min PCO_2_ difference	20.7 (13.4–28.1)	29.1 (22.4–40.2)	<0.001
Hypercapnia	47 (72.3%)	62 (89.9%)	0.009
Hypocapnia	60 (92.3%)	65 (94.2%)	0.739

Data are presented as mean (SD), median (Q1, Q3) or counts (%). BPD: bronchopulmonary dysplasia; CS: cesarean section; HFOV: high-frequency oscillatory ventilation; IVH: intraventricular hemorrhage; NEC: necrotizing enterocolitis; NICU: neonatal intensive care unit; Mg: magnesium; PDA: patent ductus arteriosus; PPROM: preterm premature rupture of membranes; RDS: respiratory distress syndrome; ROP: retinopathy of prematurity; SBI: severe brain injury; SGA: small for gestational age.

**Table 4:** Multiple logistic regression models.

Death or Severe Brain Injury Model		
Predictor	OR (95%)	*p*-Value
Gestational age < 28 weeks	22.5 (8.5–68.7)	<0.001
Air-leak syndromes	4.6 (1.3–17.3)	0.018
Pulmonary hemorrhage	3.8 (1.1–15.8)	0.046
Base Deficit (absolute value)	1.2 (1–1.4)	0.022
**Severe brain injury model**		
Predictor	OR (95%)	*p*-value
Gestational age < 28 weeks	20.6 (7.7–63.7)	<0.001
Air-leak syndromes	6.2 (1.8–23.8)	0.005
Pulmonary hemorrhage	4.5 (1.4–15.8)	0.012
Male sex	2.6 (1–7.1)	0.052
**BPD model for survivors**		
Predictor	OR (95%)	*p*-value
Gestational age < 28 weeks	9.2 (1.4–182.1)	0.043
Treated PDA	5.5 (1.2–39.9)	0.066
Hypercapnia	3.4 (1.1–11.5)	0.025

## Data Availability

The original contributions presented in the study are included in the article/Supplementary Material; further inquiries can be directed to the corresponding author.

## Supplementary Materials

The following supporting information can be downloaded at: https://www.mdpi.com/article/10.3390/jpm16040212/s1, Table S1: Perinatal and neonatal characteristics in infants with and without SBI; Table S2: Perinatal and neonatal characteristics in survivors with and without any BPD.

## Abbreviations

The following abbreviations are used in this manuscript: PCO_2_: partial pressure of carbon dioxide; PVL: periventricular leukomalacia; GA: gestational age; VG: volume guarantee; HFOV: high-frequency oscillatory ventilation; BPD: bronchopulmonary dysplasia; CS: cesarean section; IVH: intra-ventricular hemorrhage; NEC: necrotizing enterocolitis; NICU: neonatal intensive care unit; Mg: magnesium; PDA: patent ductus arteriosus; PPROM: preterm premature rupture of membranes; RDS: respiratory distress syndrome; ROP: retinopathy of prematurity; SBI: severe brain injury; SGA: small for gestational age; AIC: Akaike Information Criterion.

## References

[1] Norman M., Jonsson B., Söderling J., Björklund L.J., Håkansson S. (2023). Patterns of Respiratory Support by Gestational Age in Very Preterm Infants. Neonatology.

[2] Mukerji A., Shah P.S., Kadam M., Borhan S., Razak A. (2025). Non-invasive respiratory support in preterm infants as primary mode: A network meta-analysis. Cochrane Database Syst. Rev..

[3] Sarafidis K., Chotas W., Agakidou E., Karagianni P., Drossou V. (2021). The Intertemporal Role of Respiratory Support in Improving Neonatal Outcomes: A Narrative Review. Children.

[4] Wong S.K., Chim M., Allen J., Butler A., Tyrrell J., Hurley T., McGovern M., Omer M., Lagan N., Meehan J. (2022). Carbon dioxide levels in neonates: What are safe parameters?. Pediatr. Res..

[5] van Kaam A.H., De Jaegere A.P., Rimensberger P.C., Neovent Study Group (2013). Incidence of hypo- and hyper-capnia in a cross-sectional European cohort of ventilated newborn infants. Arch. Dis. Child. Fetal Neonatal Ed..

[6] Bayoumi El Sebaie D., Alsharany Abuelhamd W., Mohamed Abdelmomen A., Fawzy Kamal A. (2024). Is hyperventilation a common iatrogenic problem in the neonatal intensive care unit?. Childs Health.

[7] Thome U.H., Ambalavanan N. (2009). Permissive hypercapnia to decrease lung injury in ventilated preterm neonates. Semin. Fetal Neonatal Med..

[8] Brown M.K., Poeltler D.M., Hassen K.O., Lazarus D.V., Brown V.K., Stout J.J., Rich W.D., Katheria A.C. (2018). Incidence of Hypocapnia, Hypercapnia, and Acidosis and the Associated Risk of Adverse Events in Preterm Neonates. Respir. Care.

[9] Kaiser J.R., Gauss C.H., Pont M.M., Williams D.K. (2006). Hypercapnia during the first 3 days of life is associated with severe intraventricular hemorrhage in very low birth weight infants. J. Perinatol. Off. J. Calif. Perinat. Assoc..

[10] Fabres J., Carlo W.A., Phillips V., Howard G., Ambalavanan N. (2007). Both extremes of arterial carbon dioxide pressure and the magnitude of fluctuations in arterial carbon dioxide pressure are associated with severe intraventricular hemorrhage in preterm infants. Pediatrics.

[11] Erickson S.J., Grauaug A., Gurrin L., Swaminathan M. (2002). Hypocarbia in the ventilated preterm infant and its effect on intraventricular haemorrhage and bronchopulmonary dysplasia. J. Paediatr. Child Health.

[12] Ozawa Y., Miyake F., Isayama T. (2022). Efficacy and safety of permissive hypercapnia in preterm infants: A systematic review. Pediatr. Pulmonol..

[13] Travers C.P., Gentle S.J., Shukla V.V., Aban I., Yee A.J., Armstead K.M., Benz R.L., Laney D., Ambalavanan N., Carlo W.A. (2025). Late Permissive Hypercapnia for Mechanically Ventilated Preterm Infants: A Randomized Trial. Pediatr. Pulmonol..

[14] Altaany D., Natarajan G., Gupta D., Zidan M., Chawla S. (2015). Severe Intraventricular Hemorrhage in Extremely Premature Infants: Are high Carbon Dioxide Pressure or Fluctuations the Culprit?. Am. J. Perinatol..

[15] Pierrat V., Duquennoy C., van Haastert I.C., Ernst M., Guilley N., de Vries L.S. (2001). Ultrasound diagnosis and neurodevelopmental outcome of localised and extensive cystic periventricular leucomalacia. Arch. Dis. Child. Fetal Neonatal Ed..

[16] Papile L.A., Burstein J., Burstein R., Koffler H. (1978). Incidence and evolution of subependymal and intraventricular hemorrhage: A study of infants with birth weights less than 1500 gm. J. Pediatr..

[17] Jobe A.H., Bancalari E. (2001). Bronchopulmonary dysplasia. Am. J. Respir. Crit. Care Med..

[18] Röhr M., Poryo M., Bay J., Gortner L., Meyer S. (2017). Episodes of hypo- and hypercapnia in a cohort of mechanically ventilated VLBW infants: The role of adequate staffing. Wien. Med. Wochenschr..

[19] Murthy P., Zein H., Thomas S., Scott J.N., Mehrem A.A., Esser M.J., Lodha A., Metcalfe C., Kowal D., Irvine L. (2020). Neuroprotection Care Bundle Implementation to Decrease Acute Brain Injury in Preterm Infants. Pediatr. Neurol..

[20] Thome U.H., Dreyhaupt J., Genzel-Boroviczeny O., Bohnhorst B., Schmid M., Fuchs H., Rohde O., Avenarius S., Topf H.-G., Zimmermann A. (2018). Influence of PCO_2_ Control on Clinical and Neurodevelopmental Outcomes of Extremely Low Birth Weight Infants. Neonatology.

[21] Levene M. (2007). Minimising neonatal brain injury: How research in the past five years has changed my clinical practice. Arch. Dis. Child..

[22] Zhou W., Liu W. (2008). Hypercapnia and hypocapnia in neonates. World J. Pediatr..

[23] Fritz K., Sanidas G., Cardenas R., Ghaemmaghami J., Byrd C., Simonti G., Valenzuela A., Valencia I., Delivoria-Papadopoulos M., Gallo V. (2024). Hypercapnia Causes Injury of the Cerebral Cortex and Cognitive Deficits in Newborn Piglets. eNeuro.

[24] Sweet D.G., Carnielli V.P., Greisen G., Hallman M., Klebermass-Schrehof K., Lavizzari A., Ozek E., Pas A.T., Roehr C.C., Saugstad O.D. (2026). European Consensus Guidelines on the Management of Respiratory Distress Syndrome—2025. Neonatology.

[25] Thome U.H., Genzel-Boroviczeny O., Bohnhorst B., Schmid M., Fuchs H., Rohde O., Avenarius S., Topf H.-G., Zimmermann A., Faas D. (2015). Permissive hypercapnia in extremely low birthweight infants (PHELBI): A randomised controlled multicentre trial. Lancet Respir. Med..

[26] Shah P.S., Lui K., Sjörs G., Mirea L., Reichman B., Adams M., Modi N., Darlow B.A., Kusuda S., Feliciano L.S. (2016). Neonatal Outcomes of Very Low Birth Weight and Very Preterm Neonates: An International Comparison. J. Pediatr..

[27] Razak A., Johnston E., Stewart A., Clark M.A.T., Stevens P., Charlton M., Wong F., McDonald C., Hunt R.W., Miller S. (2024). Temporal Trends in Severe Brain Injury and Associated Outcomes in Very Preterm Infants. Neonatology.

[28] Lee J., Lee C.Y.M., Naiduvaje K., Wong Y., Bhatia A., Ereno I.L., Ho S.K.Y., Yeo C.L., Rajadurai V.S. (2023). Trends in neonatal mortality and morbidity in very-low-birth-weight (VLBW) infants over a decade: Singapore national cohort study. Pediatr. Neonatol..

[29] Subramanian S., El-Mohandes A., Dhanireddy R., Koch M.A. (2011). Association of bronchopulmonary dysplasia and hypercarbia in ventilated infants with birth weights of 500–1499 g. Matern. Child. Health J..

[30] Dassios T. (2023). On carbon dioxide and bronchopulmonary dysplasia. Acta Paediatr..

[31] Liu W., Zong H., Jiang J., Yang C., Li F. (2025). High-frequency oscillatory ventilation with volume guarantee in infants: A systematic review. Pediatr. Res..

[32] Solberg M.T., Bjørk I.T., Hansen T.W.R. (2013). Adherence to oxygenation and ventilation targets in mechanically ventilated premature and sick newborns: A retrospective study. BMC Pediatr..

[33] Nadeem M., Murray D., Boylan G., Dempsey E.M., Ryan C.A. (2010). Blood carbon dioxide levels and adverse outcome in neonatal hypoxic-ischemic encephalopathy. Am. J. Perinatol..

[34] Canadian Paediatric Society Neuroprotection from Acute Brain Injury in Preterm Infants. https://cps.ca/en/documents/position/neuroprotection.

[35] Sweet D.G., Carnielli V.P., Greisen G., Hallman M., Klebermass-Schrehof K., Ozek E., Pas A.T., Plavka R., Roehr C.C., Saugstad O.D. (2023). European Consensus Guidelines on the Management of Respiratory Distress Syndrome: 2022 Update. Neonatology.

[36] Sweet D.G., Carnielli V., Greisen G., Hallman M., Ozek E., Pas A.T., Plavka R., Roehr C.C., Saugstad O.D., Simeoni U. (2019). European Consensus Guidelines on the Management of Respiratory Distress Syndrome—2019 Update. Neonatology.

[37] de Bijl-Marcus K., Brouwer A.J., De Vries L.S., Groenendaal F., Wezel-Meijler G.v. (2020). Neonatal care bundles are associated with a reduction in the incidence of intraventricular haemorrhage in preterm infants: A multicentre cohort study. Arch. Dis. Child. Fetal Neonatal Ed..

[38] Erni I., Bassler D., Glauser D., Wolff M., Grass B., Adams M. (2025). Quality improvement project to reduce intraventricular haemorrhage in very preterm infants failed due to increased life-sustaining intensive care at low gestational age. BMJ Open Qual..

