# Supplementary material for: Early Postnatal Hypocapnia and Hypercapnia in Ventilated Preterm Infants: Incidence and Associations with Adverse Outcomes
Source: J Pers Med. 2026 Apr 12;16(4):212. doi: 10.3390/jpm16040212 (PMC13118081; doi:10.3390/jpm16040212)
Supplement: Supplementary file 1 [file jpm-16-00212-s001.zip › TABLE S1.pdf]

**Table S1**. Perinatal and neonatal characteristics in infants with and without SBI.

| Variable | Without SBI ($n$=77) | With SBI ($n$=57) | $p$-value |
|---|---|---|---|
| Gestational age (weeks) | 29 (28; 30) | 25 (24; 26) | <0.001 |
| Gestational age < 28 weeks | 15 (19.5%) | 47 (82.5%) | <0.001 |
| Birth weight (g) | 1130 (880; 1350) | 710 (610; 960) | <0.001 |
| 5-min Apgar score | 8 (7; 9) | 7 (7; 8) | 0.029 |
| Male sex | 30 (39.0%) | 33 (57.9%) | 0.030 |
| SGA | 15 (19.5%) | 5 (8.8%) | 0.094 |
| Maternal hypertension | 16 (20.8%) | 6 (10.5%) | 0.113 |
| Chorioamnionitis | 15 (19.5%) | 13 (22.8%) | 0.640 |
| PPROM | 16 (20.8%) | 19 (33.3%) | 0.102 |
| Prenatal steroids | 65 (84.4%) | 51 (89.5%) | 0.396 |
| Mg administration | 42 (54.5%) | 34 (59.6%) | 0.556 |
| Caesarean section | 69 (89.6%) | 45 (78.9%) | 0.087 |
| Inborn | 70 (90.9%) | 50 (87.7%) | 0.551 |
| Surfactant for RDS | 68 (88.3%) | 55 (96.5%) | 0.116 |
| Pulmonary hemorrhage | 7 (9.1%) | 24 (42.1%) | <0.001 |
| Air-leak syndromes | 9 (11.7%) | 16 (28.1%) | 0.016 |
| Treated PDA | 19 (24.7%) | 35 (61.4%) | <0.001 |
| Sepsis (Culture positive; early/late) | 37 (48.1%) | 32 (56.1%) | 0.354 |
| Fentanyl or sedative | 34 (44.2%) | 35 (61.4%) | 0.048 |
| NEC (grade II, III) | 8 (10.4%) | 9 (15.8%) | 0.353 |
| Any BPD | 42 (54.5%) | 16 (28.1%) | 0.002 |
| Treated ROP | 6 (7.8%) | 8 (14.0%) | 0.243 |
| Rescue HFOV | 30 (39.0%) | 39 (68.4%) | 0.001 |
| Duration of invasive ventilation | 4 (2; 11) | 6 (3; 13) | 0.098 |
| Survival | 65 (84.4%) | 18 (31.6%) | <0.001 |
| Length of stay (days) | 63 (44; 95) | 11 (7; 58) | <0.001 |
| At NICU admission | | | |
| pH | 7.3 (7.3; 7.4) | 7.3 (7.2; 7.4) | 0.581 |
| $PCO_2$ | 37.6 (31.3; 45.7) | 36.1 (29.4; 42.1) | 0.173 |
| Base Deficit (absolute value) | 6.6 (4.7; 8.9) | 7.2 (5.9; 9.5) | 0.058 |
| During the first 3 days of life | | | |
| Min $PCO_2$ | 28.6 (± 5.4) | 27.3 (± 4.9) | 0.137 |
| Max $PCO_2$ | 53.1 (± 13.1) | 59.1 (± 13.1) | 0.010 |
| Max-min $PCO_2$ difference | 21.9 (13.6; 29.5) | 29.9 (22.5; 39.6) | 0.001 |
| Hypercapnia | 58 (75.3%) | 51 (89.5%) | 0.038 |
| Hypocapnia | 70 (90.9%) | 55 (96.5%) | 0.300 |

Data are presented as mean (SD), median (Q1, Q3) or counts (%).

*BPD: bronchopulmonary dysplasia; CS: caesarean section; HFOV: High frequency oscillatory ventilation; NEC: necrotizing enterocolitis; NICU: neonatal intensive care unit; Mg: magnesium; PDA: patent ductus arteriosus; PPROM: preterm premature rupture of membranes; RDS: respiratory distress syndrome; ROP: retinopathy of prematurity; SBI: severe brain injury; SGA: small for gestational age.*
