# Supplementary material for: Early Postnatal Hypocapnia and Hypercapnia in Ventilated Preterm Infants: Incidence and Associations with Adverse Outcomes
Source: J Pers Med. 2026 Apr 12;16(4):212. doi: 10.3390/jpm16040212 (PMC13118081; doi:10.3390/jpm16040212)
Supplement: Supplementary file 1 [file jpm-16-00212-s001.zip › TABLE S2.pdf]

Table S2. Perinatal and neonatal characteristics in survivors with and without any BPD.

**Variable**	**Without BPD (*n*=28)**	**With any grade BPD (*n*=55)**	***p*-value**
Gestational age (weeks)	30 (29; 31)	28 (26; 29)	<0.001
Gestational age < 28	1 (3.6%)	21 (38.2%)	<0.001
Birth weight (g)	1318 (1122; 1615)	1070 (852; 1205)	<0.001
5-min Apgar score	8 (7; 8)	8 (7; 9)	0.830
Male sex	9 (32.1%)	27 (49.1%)	0.141
SGA	2 (7.1%)	9 (16.4%)	0.319
Maternal hypertension	4 (14.3%)	12 (21.8%)	0.559
Chorioamnionitis	3 (10.7%)	13 (23.6%)	0.240
PPROM	5 (17.9%)	12 (21.8%)	0.779
Prenatal steroids	22 (78.6%)	47 (85.5%)	0.428
Mg administration	13 (46.4%)	31 (56.4%)	0.391
Caesarean section	26 (92.9%)	50 (90.9%)	0.999
Inborn	24 (85.7%)	53 (96.4%)	0.173
Surfactant for RDS	26 (92.9%)	50 (90.9%)	1.000
Pulmonary hemorrhage	2 (7.1%)	6 (10.9%)	0.711
Air-leak syndromes	1 (3.6%)	9 (16.4%)	0.153
Treated PDA	2 (7.1%)	24 (43.6%)	0.001
Severe IVH	1 (3.6%)	5 (9.1%)	0.658
SBI	4 (14.3%)	14 (25.5%)	0.277
Sepsis (Culture positive; early/late)	11 (39.3%)	34 (61.8%)	0.051
Fentanyl or sedative	7 (25.0%)	31 (56.4%)	0.007
NEC (grade II, III)	1 (3.6%)	9 (16.4%)	0.153
Treated ROP	1 (3.6%)	13 (23.6%)	0.028
Rescue HFOV	4 (14.3%)	23 (41.8%)	0.013
Duration of invasive ventilation	2 (1; 3)	6 (3; 17)	<0.001
Length of stay (days)	54 (44; 67)	95 (69; 130)	<0.001
At NICU admission			
pH	7.3 (7.3; 7.4)	7.3 (7.2; 7.4)	0.567
PCO_2	34.9 (30.0; 49.1)	38.0 (32.7; 44.9)	0.323
Base Deficit (absolute value)	6.3 (± 2.8)	6.6 (± 3.0)	0.754
During the first 3 days of life			
Min PCO_2	28.6 (± 5.5)	28.8 (± 5.4)	0.891
Max PCO_2	47.1 (± 7.2)	52.9 (± 11.4)	0.006
Max-min PCO_2 difference	16.9 (12.0; 25.0)	22.1 (13.6; 31.3)	0.075
Hypercapnia	16 (57.1%)	44 (80.0%)	0.028
Hypocapnia	25 (89.3%)	52 (94.5%)	0.400

Data are presented as mean (SD), median (Q1, Q3) or counts (%).

BPD: bronchopulmonary dysplasia; CS: caesarean section; HFOV: High frequency oscillatory ventilation; IVH: Intraventricular hemorrhage; NEC: necrotizing enterocolitis; NICU: neonatal intensive care unit; Mg: magnesium; PDA: patent ductus arteriosus; PPROM: preterm premature rupture of membranes; RDS: respiratory distress syndrome; ROP: retinopathy of prematurity; SBI: severe brain injury; SGA: small for gestational age.
